# The GABA_B_ Receptor—Structure, Ligand Binding and Drug Development

**DOI:** 10.3390/molecules25133093

**Published:** 2020-07-07

**Authors:** Linn Samira Mari Evenseth, Mari Gabrielsen, Ingebrigt Sylte

**Affiliations:** Molecular Pharmacology and Toxicology, Department of Medical Biology, Faculty of Health Sciences, UiT The Arctic University of Norway, NO-9037 Tromsø, Norway; mari.gabrielsen@uit.no (M.G.); ingebrigt.sylte@uit.no (I.S.)

**Keywords:** GABA_B_ receptors, orthosteric binding site, allosteric binding site, structural mechanisms, drug development, baclofen

## Abstract

The γ-aminobutyric acid (GABA) type B receptor (GABA_B_-R) belongs to class C of the G-protein coupled receptors (GPCRs). Together with the GABA_A_ receptor, the receptor mediates the neurotransmission of GABA, the main inhibitory neurotransmitter in the central nervous system (CNS). In recent decades, the receptor has been extensively studied with the intention being to understand pathophysiological roles, structural mechanisms and develop drugs. The dysfunction of the receptor is linked to a broad variety of disorders, including anxiety, depression, alcohol addiction, memory and cancer. Despite extensive efforts, few compounds are known to target the receptor, and only the agonist baclofen is approved for clinical use. The receptor is a mandatory heterodimer of the GABA_B1_ and GABA_B2_ subunits, and each subunit is composed of an extracellular Venus Flytrap domain (VFT) and a transmembrane domain of seven α-helices (7TM domain). In this review, we briefly present the existing knowledge about the receptor structure, activation and compounds targeting the receptor, emphasizing the role of the receptor in previous and future drug design and discovery efforts.

## 1. Introduction

The metabotropic γ-aminobutyric acid (GABA) type B receptor (GABA_B_-R) was first described in 1979 by Dr. Norman Bowery and was acknowledged to play an important inhibitory role in neurotransmission [1,2,3]. The receptor was not cloned successfully until 20 years later because of the lack of high-affinity radioligands and, more importantly, due to the unexpected structural features of the receptor [4]. The GABA_B_-R belongs to class C of G-protein coupled receptors (GPCRs) together with the metabotropic glutamate receptors (mGluRs), calcium-sensing (CaS), taste 1 and orphan receptors [5]. Until recently, only the structure of the extracellular Venus flytrap (VFT) domain hosting the orthosteric binding was known from X-ray crystallography studies. However, recently, four papers were published, describing the cryogenic electron microscopy (cryo-EM) structures of the full-length receptor in several conformations [6,7]. The paper by Mao et al. [6] describes both a full-length active conformation bound to the agonist baclofen and the positive allosteric modulator (PAM) (R,S)-5,7-di-*tert*-butyl-3-hydroxy-3-trifluoromethyl-3H-benzofuran-2-one (BHFF) in the presence of the G_i1_-protein, and a full-length inactive antagonist (CGP54626)-bound receptor [6]. The paper by Shaye and coworkers [7] describes the full-length inactive apo receptor, an active receptor conformation bound to the agonist SKF97541 and the PAM GS39783, and two intermediate agonist-bound receptor conformations. The latest papers by Papasergi-Scott et al. and Park et al. describes the cryo-EM structure of the full-length inactive receptor [8,9]. These structures may provide new opportunities for understanding receptor mechanisms and for drug discovery.

Targeting GABA_B_-R activity is considered a valuable therapeutic approach in several neurological and psychiatric disorders including depression, schizophrenia, addiction and cancer [10,11,12]. During the years, large efforts have been devoted to develop GABA_B_-R compounds for therapeutic use [13,14,15,16]. However, obtaining GABA_B_-R compounds with favorable pharmacokinetics, desired effects, and tolerable side effects has proven to be difficult. The only approved drug targeting the GABA_B_-R is baclofen, which is used as a muscle relaxant and antispastic agent [17,18]. Baclofen has multiple pharmacokinetic limitations including low blood-brain barrier (BBB) penetration, short duration of action and rapid tolerance developments in the patients [19,20].

The present review is focusing on the structure and function of the GABA_B_-R, the interactions of the receptor with known orthosteric and allosteric compounds and development of new compounds. Despite great efforts in drug design and discovery conducted both by the academia and industry, dating decades back, only few ligands are known to target the GABA_B_-R including agonists, antagonists and allosteric modulators.

## 2. Structure of the GABA_B_ Receptor

**Figure 1:**
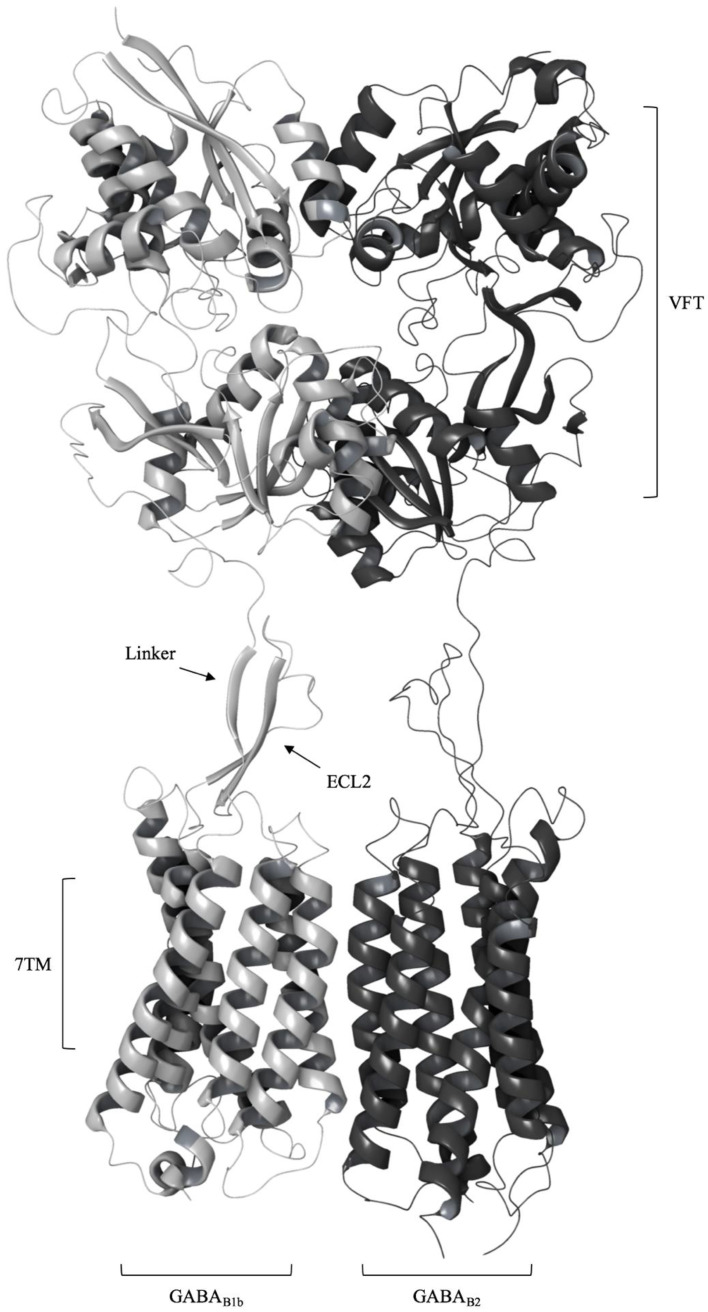
A schematic representation of the (GABA) type B receptor (GABA_B_-R) heterodimer with the extracellular Venus Flytrap domain (VFT) and the heptahelical transmembrane domain (7TM) of the GABA_B1b_ (gray) and GABA_B2_ (black) protomers. The VFT is connected to the 7TM by a linker that interacts with the extracellular loop 2 (ECL2) of 7TMs (active receptor conformation (Protein Data Bank (PDB) ID: 6OU8).

The GABA_B_-R is an obligate heterodimer composed of the GABA_B1_ and GABA_B2_ subunits. Each subunit consists of an extracellular VFT domain linked to a heptahelical transmembrane (7TM) domain, and the linker is shorter in sequence and lacks the cysteine residues that are conserved among other class C GPCRs (called the cysteine rich domain (CRD)) (Figure 1) [21]. Radioligand binding studies, site-directed mutagenesis studies and the X-ray crystal structures showed that the orthosteric binding site of GABA_B_-R is located in the VFT of GABA_B1_, while ligand binding to the GABA_B2_ VFT have not been observed [22]. Binding studies of isolated GABA_B2_ subunits and studies manipulating the receptor composition showed that the GABA_B2_ 7TM domain is mainly responsible for recruiting G-proteins, in addition to hosting an allosteric binding site [23,24,25]. There is also evidence from biochemical and biophysical studies that the GABA_B1_ 7TM participates in formation of GABA_B_-R oligomers by interacting with other GABA_B1_ 7TMs in the inactive state [26,27,28]. The receptor was found to be in equilibrium between heterodimers and oligomers by applying SNAP-tag technology [27]. The formation of oligomers represents a new level of complexity as the functional consequence can be the transactivation of receptors, which is currently only established for heterodimers [29].

There are multiple isoforms of the GABA_B1_ subunit, but the most abundant are the GABA_B1a_ and GABA_B1b_, encoded by the same gene, GABBR1 [4]. Structurally they only differ in the N-terminal region with the presence of two so-called sushi domain repeats, sushi 1 and sushi 2, located on the GABA_B1a_ subunit [30]. The sushi domains are reported to function as an intracellular sorting signal responsible for trafficking the isoform into axons [30], and has not been implicated to affect the receptor pharmacology or kinetics in heterologous cells [31,32]. However, a previous study showed that the sushi 1 domain can bind to the secreted ectodomain of amyloid-β precursor protein associated (APP) which is associated with Alzheimer’s disease, and thereby suppress synaptic vesicle release [33]. The intracellular C-terminal regions interact by forming a coiled-coil domain stabilizing the heterodimerization. The C-terminal coiled-coil region constitutes two parallel helices with five heptad repeats [34]. GABA_B1_ is dependent on dimerization with GABA_B2_ for trafficking from the endoplasmic reticulum (ER) to the cell surface as GABA_B2_ masks a retention signal which is present in the end of the coiled-coil domain of GABA_B1_ [34]. The C-terminal GABA_B_-R region also serves as binding site for multiple proteins associated with regulation of the receptor such as the potassium channel tetramerization domain-containing proteins (KCTDs) [35].

### 2.1. The Extracellular Domains

**Figure 2:**
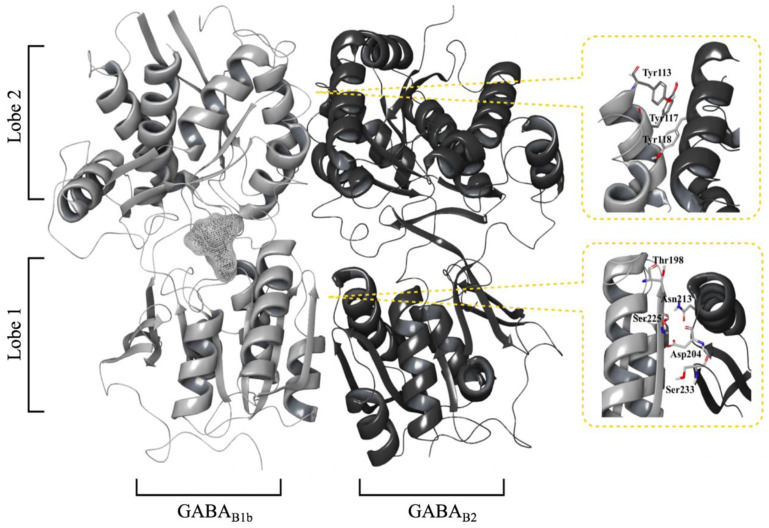
The heterodimeric extracellular GABA_B1b_ receptor VFTs in the active state (PDB ID: 4MS4). Residues important for the Lobe 1-Lobe 1 and Lobe 2-Lobe 2 interactions are displayed in the yellow boxes. The approximate position of the binding pocket is displayed as a grey mesh in GABA_B1_.

Nine X-ray crystal structures of the GABA_B_-R VFT dimer (GABA_B1_ and GABA_B2_ VFTs) in complex with various ligands are available in the Protein Data Bank (PDB), two structures with different agonists, six with different antagonists and one apo conformation [36]. The X-ray structures show that agonist binding is associated with a closed/active conformation of the GABA_B1_ VFT, while antagonist binding is associated with an open/inactive conformation. The X-ray structures also show that the VFT heterodimer is formed by non-covalent interactions between GABA_B1_ and GABA_B2_ (Figure 2). Each VFT contains two distinct lobes, the N-terminal Lobe 1 (LB1) and the C-terminal Lobe 2 (LB2) [36], where LB1 of GABA_B1a/b_ VFT interacts with LB1 of GABA_B2_ VFT both in the active and inactive conformational states. This interface is fully facilitated by non-covalent interactions which involves patches of hydrophobic interactions, hydrogen bonds and a salt bridge [37]. The hydrophobic interactions are conserved among all available GABA_B_-R VFT X-ray structures and are mainly facilitated by three tyrosine residues [36]. The three tyrosine residues are stacking at the LB1-LB1 interface, and site-directed mutagenesis studies (from Tyr to Ala) proved that they are important for agonist-dependent G_i_-protein activation and the GABA-induced current of G-protein coupled inward rectifying potassium channels (GIRKs) [37,38]. The single-point mutations significantly decreased GABA-induced stimulation of [^35^S]GTP-γS binding compared to wild type, but had no effect on the GABA affinity [37]. Receptor activation induces large conformational changes such that the LB2 domains also form a large heterodimer interface, which is facilitated by a network of hydrogen bonds (Figure 2) [37].

Sequence analysis showed that none of the residues associated with ligand binding in the GABA_B1b_ VFT are conserved in the GABA_B2_ VFT [37], and the sequence homology between the VFTs is 33% [22]. X-ray structures in complex with agonists show that the GABA_B2_ VFT remains in an open state in spite of the agonist-induced closed conformation of the GABA_B1a_ VFT [36]. Moreover, binding studies with recombinant receptor mutants indicated that the VFT of GABA_B1_ is functional in absence of the GABA_B2_ VFT [39,40]. However, the GABA_B2_ VFT is suggested to impact receptor activation by promoting signal transduction from the extracellular side to the intracellular side, and contribute to increased agonist affinity and efficacy [39,40].

Single-molecule Förster resonance energy transfer (smFRET) studies of mGluRs showed that the VFTs oscillate between the open/inactive and closed/active conformation and that binding of an agonist shifts the conformational equilibrium towards the closed state [41,42]. This mechanism has not been confirmed for the GABA_B_-R, and conformational rearrangements associated with activation of the GABA_B_-R are found to be smaller than what is observed for mGluRs [6,36,43,44]. The open/inactive and closed/active states of GABA_B_-R are likely to be the main conformational states of the VFTs as seen in the X-ray structures, and were found to be iso-energetic by molecular dynamic studies in combination with enhanced sampling, while intermediate conformations were found in higher energetic states [45].

The linker region between the VFT and 7TM in both subunits consists of approximately 20 residues and was earlier described as non-critical for GABA_B_-R activation and signal transduction from the GABA_B1_ VFT to the 7TM domains as seen by modifying the linkers [46,47]. The distance between the C-terminus of the two LB2 subunits decreases from approximately 45 to 32 Å upon activation [36], and the recently released cryo-EM structures from Shaye et.al show that the linker interacts extensively with the extracellular loop 2 (ECL2) of the 7TMs through an anti-parallel β-sheet, and is thereby suggested to contribute to stabilize the active conformation of the 7TM domains [7]. Interactions between the ECL2 and the CRD are also described for mGlu5, and mutational studies showed that the deletion of certain residues in ECL2 reduced the agonists’ efficacy [44].

### 2.2. Transmembrane Domains

Changes in the 7TM subunit interaction interface upon agonist activation of the GABA_B_-R have been quantified by cysteine cross-linking studies [26], and is also described in the in the newly released papers describing cryo-EM structures [6,7]. The 7TM domain interface interactions in the inactive state is formed by TM3 (GABA_B1_)–TM5 (GABA_B2_) and TM5 (GABA_B1_)–TM3 (GABA_B2_) interactions and that the interactions between the protomers are facilitated through ionic interactions stabilized by aromatic residues [6,9,26]. The distance between the backbones of TM5 in each monomer is 8 Å and thereby much shorter than the distance between the homodimers in the inactive mGlu5, which is 21 Å [6,44]. Cross-linking studies and the cryo-EM structures indicate that activation induces a rotation of the domains, forming a new interface consisting of TM6 of both monomers as also described for mGlu5 [6,26,44].

As previously described, binding studies and manipulations of the receptor composition have acknowledged the GABA_B2_ 7TM domain as mainly responsible for recruiting G-proteins [23,24,25]. One of the recent cryo-EM structures described by Mao et al. indicates that the GABA_B1_ may also couple to G-proteins as they identified a thermostable conformation where this occurred [6]. Mao et al. further suggest that G-proteins may bind to one of the subunits at a time due to sterically hindrance, favoring the GABA_B2_ as this was the most frequent populated distribution found in processing the cryo-EM data [6]. The recent cryo-EM structures describe that the extracellular half of the 7TM region of both monomers are occupied by a phospholipid, in both the active and inactive conformations, at a site corresponding to the orthosteric binding sites in most family A GPCRs [6,8,9]. Moreover, a “lid” is formed over the lipids in each subunit by residues located in the ECL2 of GABA_B1b_, and all ECLs in GABA_B2_ including the linker [8,9]. The lipids are suggested to play a role in receptor activation and structural integrity as seen by mutational studies of interacting residues in GABA_B1b_, trying to displace the lipid tail and interfere with coordination of the head group [8,9]. The presence of the lipid also blocks the access of allosteric modulators to the 7TM domains, which would have to displace the lipid or bind to an alternative binding site [8]. Park et al. suggests that the lipid in GABA_B1b_ acts as a negative allosteric modulator (NAM) by stabilizing the inactive conformation [9]. We must note that lipids were not present in the recently released structures by Shaye et al. [7].

The 7TM helices are connected by three intracellular and three extracellular loops as seen for all GPCRs [21]. Loop-swapping and mutational studies have identified the second and, especially, the third intracellular loop as important in G-protein binding [48,49].

## 3. GABA_B_ Receptor Binding Sites

### 3.1. Orthosteric Binding Site

The orthosteric binding pocket is located in the crevice of LB1 and LB2 of GABA_B1a/b_. Binding of agonist induces large conformational changes such that the LB1 and LB2 interact and form a stable closed conformation in timescales necessary for full receptor activation (Figure 2) [36,50].

**Figure 3:**
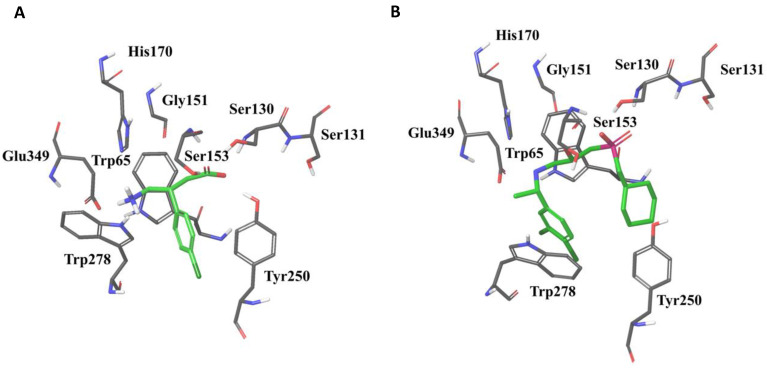
The agonist baclofen (**A**) and the antagonist CGP54626 (**B**) in the GABA_B1b_ VFT binding pocket surrounded with residues important for ligand binding (PDB ID: 4MS4 and 4MR7, respectively). Trp278 and Tyr250 are located in Lobe 2 of the GABA_B1_ VFT, whereas the remaining residues are located in Lobe 1.

Residues located in LB1 are responsible for anchoring both agonists and antagonists in the binding pocket (Figure 3) and their interaction patterns with residues in LB1 are highly similar [36]. Mutational studies followed by radioligand- and [^35^S]GTPγS-binding assays found that mutations of tryptophan (Trp65) and histidine (His170) of GABA_B1b_ abolished antagonist binding [36]. Agonist activation was also abolished after mutating Trp65Ala, while the His170Ala mutation only reduced receptor activation [36]. Additional mutational studies followed by radioligand binding assays showed that mutating Ser130 to Ala abolished binding of the antagonist CGP54626, while mutation of Ser153 were found to affect the affinity of various ligands differently and are thereby suggested to play a role in selectivity of GABA_B_ ligand recognition [51]. For more details about ligand interactions in the orthosteric binding site, please see [36,51,52,53].

Ligand interactions with residues located in LB2 seem to be restricted to agonists and high-affinity antagonists [36,52,53]. Interactions with residues in both LB1 and LB2 are likely to be a requirement for activation, and cause the agonists to become buried within the closed receptor conformation (Figure 3). This is supported by mutational studies indicating that Trp278 and Tyr250 located in LB2 of GABA_B1b_ are critical for agonist binding but have less effect on binding of antagonists [36,54].

All ligands co-crystalized with the VFT are structural derivatives of GABA with an α-acid and a γ-amino group [36]. The α-acid and the γ-amino groups of co-crystalized ligands are stabilized by identical residual interactions in all X-ray crystal structures, independent of intrinsic ligand activity [36,52,53]. Linking receptor interaction patterns to ligand activity and affinity has proven to be difficult, as highly similar compounds show similar receptor interaction patterns despite of different activity [36]. Larger and more bulky antagonists, like CGP54626 and CGP62349, are thought to prohibit the formation of a stable closed conformation by forming few and variable interactions with the LB2, likely as a result of the size compared to agonists (Figure 3) [36,52].

### 3.2. Allosteric Binding Site

Allosteric modulators change the efficacy and/or affinity of the orthosteric agonist [55,56]. PAMs potentiate the receptor activation induced by an orthosteric agonist, and some PAMs also display intrinsic agonist activity and are named ago-PAMs [55]. FRET studies have also shown that ago-PAMs can actually cause movements of the GABA_B1_ VFT and the 7TM, corresponding to the conformational changes observed upon agonist activation [43]. NAMs inhibit or reduce responses produced by orthosteric agonist, either by stabilizing an inactive conformation of the 7TM domain, and/or decrease the agonist affinity. Currently, less than 100 PAMs are known in the literature to target the GABA_B_-R, and there is only a single NAM to our knowledge [57,58]. Silent allosteric modulators (SAMs), also called neutral allosteric ligands (NALs), have no effect on orthosteric agonists efficacy or affinity, but can compete with other allosteric compounds and block their action [55,59]. Currently, no GABA_B_ receptor SAMs have been described in the literature.

**Figure 4:**
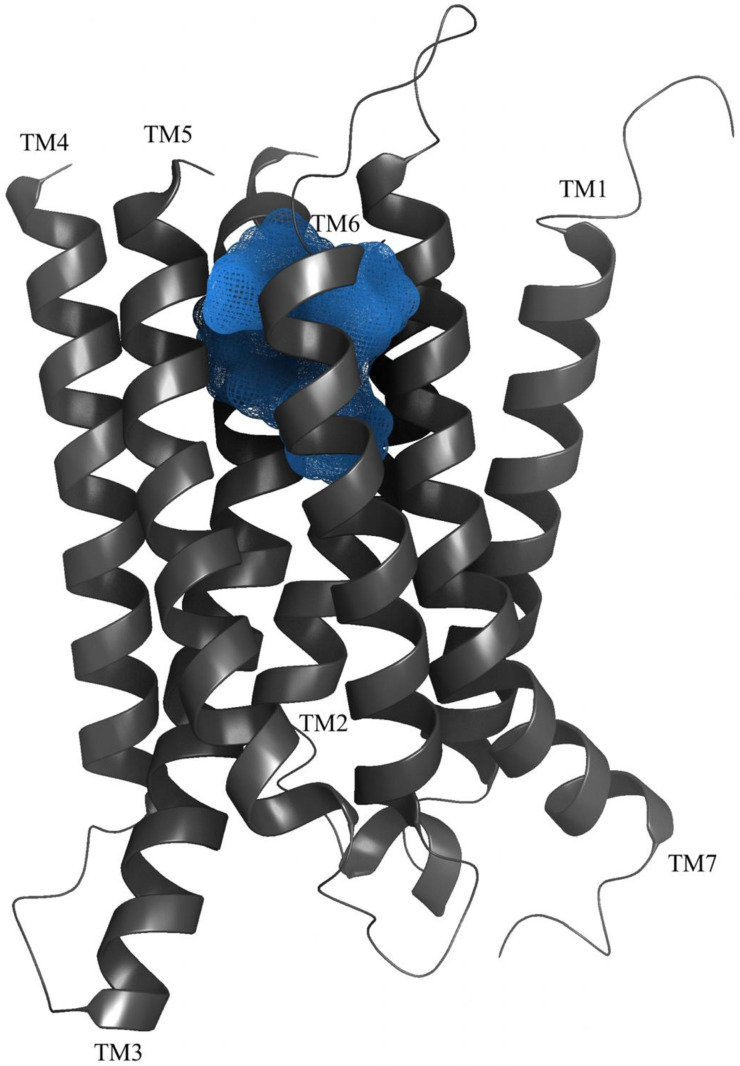
A illustration of the putative allosteric binding pocket (blue sphere) located in GABA_B2_ (dark grey) surrounded by the seven helices (TM1–TM7) (homology models obtained from Freyd et al. [57]).

Previous studies have given strong support for an allosteric binding site in 7TM domain of the GABA_B2_ subunit [60]. Binet and coworkers studied various combination of wild type and chimeric GABA_B_-R subunits and showed that the ago-PAM CGP7930 could activate GABA_B2_ expressed alone [24]. Moreover, investigations of the 7TM domain of GABA_B2_ by introducing point mutation identified the amino acids Gly706 and Ala708 in TM6 to be important for interactions with PAMs, specifically tested with the PAM GS39783 [61]. This study also showed that the PAM GS38783 could bind to a mutated rat GABA_B2_ subunit and activate the receptor without the GABA_B1_-subunit present [61]. Ligand-guided homology modelling and docking studies gave results in agreement with experimental ligand binding studies (Figure 4), and identified nine amino acids within TM3 (Tyr564 and Leu560), TM5 (Lys664), TM6 (Met702, Cys703, Gly706 and Ser710) and TM7 (Val724 and Cys731) as central for the binding of PAMs to the GABA_B2_ [57]. The binding pocket corresponds to the orthosteric binding pocket in most family A GPCRs. Allosteric modulators have been found to bind within the 7TM of other family C GPCRs as well, as seen for mGlu in multiple studies including mGlu5 crystal structures [44,62]. Mao et al. describe the cryo-EM structure of the GABA_B_-R with the ago-PAM BHFF [6], and surprisingly, a novel PAM binding pocket is located in a cavity between TM5–TM6 of GABA_B1_ and TM6 of GABA_B2_ [6]. This discovery is supported by Shaye et al., describing two binding sites for the PAM GS39783, site 1 located in the 7TM of GABA_B2_ and site 2 located at the TM6 heterodimer interface [7]. Mutational studies of these two binding sites showed that site 2 is main allosteric binding site for the PAM GS39783 [7]. Together these studies indicate that there exist at least two allosteric binding sites in the 7TM domains, one within TM3, TM5 and TM6 of the GABA_B2_ subunit, and another located in the TM6 interface of the 7TMs.

Allosteric compounds are attractive in drug development because of their ability to modulate the effect of orthosteric ligands, and thereby reduce potential side effects and/or increase desirable therapeutic effects [55]. Most allosteric modulators exert effects in company with endogenous GABA or other orthosteric ligands [63], and when used in combination with an orthosteric drug, lower doses of the orthosteric drug may be required or a specific combination might give beneficial effects due to biased signaling. However, allosteric modulators can also be effective alone, and even in reduced concentrations compared to drugs binding to the orthosteric site [64]. Designing allosteric modulators provides the possibility of making compounds that are more selective for the desired functional outcome than orthosteric compounds alone, and thereby potentially reduce the side effects [55,65]. Distinct ligand signaling profiles are caused by biased signaling, in which different ligands are understood to induce different receptor conformations, allowing potential diverse effector proteins to recognize these conformations. In theory, this can result in a variety of signaling profiles based on the specific allosteric and orthosteric compound in combination or even alone, stabilizing a certain receptor conformation [66]. Biased agonism is well characterized for class A GPCRs and class C mGluRs [67,68], and was recently described for GABA_B_-R where the PAMs GS39783 and BHF177 were found to have functional selectivity for intracellular signaling pathways in various functional assays [69].

## 4. GABA_B_ Receptor Signaling

The GABA_B_-R has a complex signaling network, and function as auto- or hetero-receptors on both inhibitory and excitatory nerve terminals. When GABA is released from a GABAergic neuron, it may inhibit further release by binding to presynaptic auto-inhibitory receptors, functioning in a negative feedback loop [70]. These auto-receptors can also be activated by GABA released by a single action potential [30]. GABA_B_-Rs are also found on non-GABAergic neurons where they act as hetero-receptors and inhibit the release of other neurotransmitters such as glutamate from glutamatergic neurons [30].

Activation of pre- and postsynaptic GABA_B_-Rs by an agonist results in the inhibition of adenylyl cyclase (AC) through the Gα_i/o_ pathway [32]. In presynaptic terminals, binding of Gα_i/o_ to AC causes decreased levels of cAMP, which prevents vesicle fusion and thereby neurotransmitter release [32]. In addition, the Gβγ subunit of the G-protein binds directly to voltage-gated Ca^2+^ channels (VGCC), resulting in inhibition of inward rectifying Ca^2+^ channels necessary for vesicle fusion [32]. The Gβγ subunit can also directly attach to SNAP receptors (SNARE) that are responsible for anchoring vesicles to the synaptic membrane and thereby inhibiting presynaptic membrane vesicle fusion [32]. In the postsynaptic membrane, the Gβγ subunit also binds to and inhibits the VGCC, but contributes to a hyperpolarization and inhibits the release of many neurotransmitters including noradrenaline, serotonin and dopamine [32]. Postsynaptically, the cAMP-dependent protein kinase A (PKA) signaling pathway is affected by the inhibition of AC [32], resulting in inhibition or reduced permeability of ion channels such as the ionotropic glutamate NMDA receptor that mediates Ca^2+^ influx [71]. In addition, the Gβγ subunit stimulates the G-protein coupled inwardly rectifying K^+^ channels (GIRK), resulting in inhibition of the postsynaptic potential and decreased long-term potentiation (LTP) [72,73]. The phosphorylation of the Extracellular Signal-Regulated Protein Kinases 1 and 2 (ERK_1/2_) in certain areas of the hippocampus, known to be important for memory and learning, has also been linked to GABA_B_-R activation [74]. ERK_1/2_ play an important role in gene expression by regulating the activity of transcription factors. A study showed that GABA and baclofen can increase the phosphorylation of ERK_1/2_ without changing the expression level in cerebellar granule neurons cultured from mouse [74]. The phosphorylation was found to be G-protein dependent as the pertussis toxin known to inhibit G_i/o_-protein coupling, also inhibited phosphorylation of ERK_1/2_ [74]. The activation of the receptor has also been linked to direct interactions with the L-type VGCC isoforms Ca_V_1.2 and Ca_V_1.3, which increase channel activity and mediate ERK_1/2_ phosphorylation via these interactions [75]. These ion channels contain multiple consensus sites for phosphorylation by protein kinases, and both phospholipase C (PLC) and protein kinase C (PKC) are suggested to be involved in GABA_B_-R-mediated facilitation of these channels [75]. The link between ERK_1/2_ and GABA_B_-Rs is as described, highly complex and additional efforts are needed to clarify the full aspect of pathway-specific activation and functional selectivity that is also likely to be cell type specific.

The C-terminal GABA_B_-R region also serves as binding site for multiple proteins, including regulatory G-protein signaling (RGS) proteins that regulate receptor activity. In addition, leucine-zipper transcription factors, scaffolding and adaptor proteins interact with the coiled-coil C-terminus of the receptor and modulate intracellular trafficking, receptor dimerization and synaptic localization contributing to the functional diversities of the GABA_B_-R [76]. GABA_B_-R signaling is also regulated by the auxiliary protein subunits, KCTDs, which control the kinetics of GIRK activation and desensitization. These effects are mediated by the binding of the KTCDs to the C-terminal of the GABA_B2_ subunit and to the Gβγ proteins [35].

## 5. GABA_B_ Receptor Pathophysiology

The disruption of GABA_B_-R signaling pathways is linked to a variety of neuropsychiatric disorders and diseases including depression, anxiety, schizophrenia, addiction, learning and memory, epilepsy, neurodegenerative disorders, cancer and gastroesophageal reflux disorder (GERD) [12,18,77,78,79,80]. The autoimmune disease Anti-GABA_B_-R encephalitis was recently described in a case report and comprises a new category of GABA_B_-R related diseases, where patients develop antibodies against the receptor [80,81]. Recent evidence suggests that neurotransmitters are involved in tumor development and proliferation of multiple cancer types [11,82]. The GABA_B_-R is found to be upregulated in a variety of cancer cell lines including hepatocellular and colon cancer cells [82]. Immunohistostaining of tumor samples from the thyroid gland found a significantly increase in GABA_B2_ expression in tumor tissue compared to normal tissue and linked the expression to malignancy [82], while increased expression of GABA_B1_ has been linked to malignancy in human breast cancer [82]. The role of GABA_B_-R activation for cell proliferation has been investigated by baclofen administration in different rat cancer models, such as gastric cancer and colon tumor models. The results showed that baclofen reduced the incident of gastric cancer significantly, and decreased the colon tumor malignancy [82]. Additional in vitro studies have displayed an inhibitory role of baclofen in tumor cell proliferation and/or migration in numerous cell lines including human pulmonary adenocarcinoma-, pancreatic duct epithelial and small airway epithelial cells. However, certain prostate cancer cell lines where not inhibited after baclofen treatment, and rather enhanced cancer migration probably by promoting matrix metalloproteinase-3 production [82].

Epilepsy is a disease caused by abnormal neural activity and is characterized by seizures [83]. There are different types of seizures depending on the neuronal network involved, and the role of GABA_B_-R depends on the type of seizure and network involved [83]. The GABA_B_-R-mediated mechanisms represent an dichotomy, as agonists might exacerbate some type of seizures and act as an anticonvulsant in other types [83]. Antagonists have also shown anticonvulsant properties in certain seizure types. The complex pathology of epilepsy, the adverse effects of GABA_B_-R agonists and the lack of appropriate and approved antagonists, indicate that treatment of this disease by targeting the GABA_B_-R must await clinical development of new ligands.

The stimulation of GABA_B_-R by baclofen is linked to a reduction in addiction-related behavior towards substances such as nicotine, cocaine and alcohol in animal models [79,84]. Drugs of abuse stimulate the mesolimbic system in the brain that controls the release of the reward-associated neurotransmitter dopamine [85]. A recent study showed that the rewarding effect of nicotine was reduced in animals pre-treated with baclofen [85]. Nicotine stimulates the nicotine acetylcholine receptors (nAchRs) located on GABAergic, glutamatergic and dopaminergic neurons, which causes release of dopamine. Baclofen activates GABA_B_-Rs in dopaminergic and GABAergic neurons, and significantly reduces the amount of dopamine release by inhibition of the dopaminergic neurons.

Baclofen and PAMs have also demonstrated anxiolytic effects in animal cognition models and have been implicated to reverse anxiogenic responses from addiction-related withdrawal [18,86,87,88]. Shortly after the first published description of the GABA_B_-R, the receptor was linked to depression [89,90], and GABA_B_-R antagonists have shown to exhibit antidepressant effects in a variety of animal models [18,86,91]. Abnormal peripheral serum concentrations of GABA and glutamate, and reduced brain levels of the enzyme glutamic acid decarboxylase (GAD), which is responsible for converting glutamate to GABA, have been found in young adults diagnosed with depression and schizophrenia [77]. Changes in GABAergic neurons and in the concentration of GABA_B_-R isoforms, have been discovered in post-mortem examination of patients diagnosed with clinical depression, and support the theory of involvement of the GABAergic system in psychiatric disorders [77,92,93].

PAMs and NAMs are under investigation for treatment of neuropsychiatric disorders [10], but currently no allosteric modulator is marketed for therapeutic use.

## 6. The GABA_B_ Receptor in Drug Discovery

**Figure 5:**
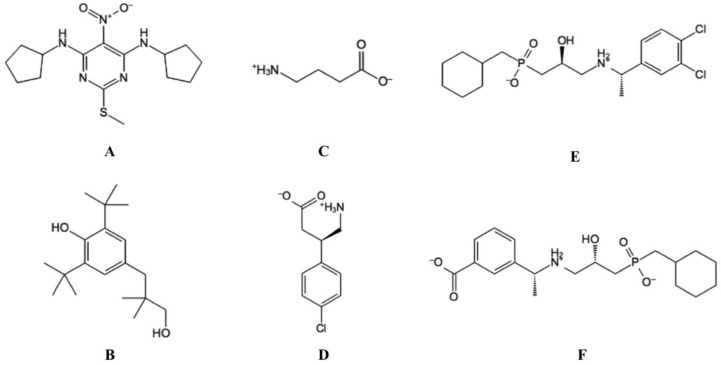
The structure of selected ligands targeting the GABA_B_-R. (**A**) GS39783, (PAM). (**B**) CGP7930, (ago-PAM). (**C**) GABA (endogenous agonist). (**D**) Baclofen (agonist), and (**E**) CGP54626 (antagonist). (**F**) CGP56999A (antagonist).

In 1967, GABA was recognized as the main inhibitory neurotransmitter in the mammalian CNS though the presence of the neurotransmitter was recognized nearly 20 years earlier [94,95]. The GABA molecule has been known for several years and was synthesized already in 1888 [94,95]. GABA is a small neurotransmitter with the molecular weight of 103 Da, has high hydrophilicity and high aqueous solubility and cannot penetrate the BBB (Figure 5). Various drug discovery and development regimes have been implemented during the last decades to find drug-like GABA analogues with more appropriate physicochemical properties for possible therapeutic application. These efforts have resulted in many GABA analogues, but, surprisingly, many of these do not bind the receptor, despite high structural similarity to GABA [96]. This emphasizes the apparent struggle of developing selective and potent ligands, but also highlight the urge for new ligands with chemotypes different from present GABA_B_-R compounds. New ligands may enhance our understanding of activation mechanisms, clarify the role of the receptor in different signaling pathways and potentially benefit in the understanding of therapeutic effects or become new drugs.

### 6.1. Orthosteric Ligands

The first known GABA derivative was baclofen, which was synthesized in 1962 by adding an halogenated phenyl ring to the β-carbon in an effort to obtain a compound that could penetrate the BBB (Figure 5) [83,97]. An investigation into baclofen showed that also this compound had multiple limitations; it could not passively penetrate the BBB and had a short half-life requiring frequent administration and thereby causing multiple side effects [83]. The endogenous precursor of GABA, γ-hydroxybutyrate (GHB) is a GABA_B_-R partial agonist and is marketed as a therapeutic drug for treatment of narcolepsy, and a frequently abused drug for recreational purposes. What connects the pharmacological effects of GHB to the GABA_B_-R is uncertain, as GHB also binds the GABA_A_ receptor and the GHB receptor [32,98].

Great efforts have been made to obtain baclofen analogues, but without producing better drug candidates. Phenibut was one of these compounds produced by simply removing the chlorine atom of baclofen [83]. The compound showed anxiolytic and nootropic effects, but was quickly identified as not selective for the GABA_B_-R [99]. Other studies aimed to rigidify the baclofen structure by introducing groups such as ethylene and propylene, but without luck [83]. However, some of the efforts in obtaining baclofen analogues actually resulted in clinically approved drugs, such as pregabalin, vigabatrin and gabapentin [83], but these were found to act through different mechanisms and bind other targets than the GABA_B_-R.

A major breakthrough in the development of a new series of potent GABA_B_-R agonists was discovered by replacement of the carboxylic acid of GABA for unsubstituted phosphinic and methylphosphinic acids [13]. Some of these compounds were found to be very potent in vitro, but unfortunately not in vivo, while others were found to function only on peripheral GABA_B_-R, like Lesogaberan [83]. A series of antagonists was also derived from GABA and baclofen by replacing the α-acid group with substituted phosphonic and sulfonic acid groups [14,15,16]. These baclofen analogues were found to be low-affinity antagonists, such as 2-hydroxysaclofen, which also was co-crystallized with the GABA_B_-R VFTs (PDB ID: 4mqf). Despite that this compound show antagonistic properties, the interaction pattern with the VFT is more similar to the interaction patterns of agonists [36]. The GABA phosphinic acids analogues were found to be high-affinity antagonists in the lower nM range when further modified by adding benzyl substituents (Figure 5) [14,100]. Some of these compounds were also developed into radioligands which were later used to identify the receptor in cloning experiments [83].

Recently, the difluoromethyl ketone scaffold was identified as a GABA_B_-R agonist scaffold, and exhibited in vivo activity in mice as potential anxiolytic drugs [101]. Difluoromethyl ketones represent a new chemotype of GABA_B_-R ligands, but need to be further investigated as many of these compounds are known to bind multiple other targets such as the matrix metalloproteases [101,102].

### 6.2. Allosteric Ligands

The first PAM, CGP7930 (Figure 5), was discovered by a high throughput screening campaign in 2001 [103]. The compound showed a dose-dependent potency and efficacy in presence of GABA, and was later proven to function as an ago-PAM [103]. The compound potentiates the sedative effects of baclofen, has antidepressant-like and anxiolytic effects in animal models, and reduces addiction related behavior towards alcohol and cocaine [16]. CGP7930 structurally resembles the anesthetic drug propofol, and was later used as a starting point to synthesize additional PAMs. A couple of years later, a new series of novel PAMs were discovered [104]. GS39783 (Figure 5) was among the most potent compounds of this series and like CGP7930, it showed anxiolytic effects and reduction in addiction-related behavior [83]. Large attempts were put into producing less genotoxic compounds, and in 2008 Addex therapeutics patented over 300 compounds identified by high-throughput screening and lead optimization [83]. In total 23 compounds showed satisfactory in vitro activity, and were further evaluated in animal models of anxiety and pain. These studies resulted in the compounds ADX71943 and ADX71441. ADX71943 showed a poor safety profile, while ADX71441 was under investigation for treatment of diseases such as Charcot-Marie-Tooth Type 1A disease and addiction until recently according to Addex Therapeutics, where they also announce that they are accelerating the GABA_B_-R PAM optimization and development [105]. In recent years, multiple efforts have been made to develop new PAMs, especially by drug companies like Roche, Addex and AstraZeneca, and though multiple compounds show activity, yet none are approved as clinical therapeutics due to failing safety profiles [83]. The first NAM was discovered in 2014 in an attempt to identify new PAMs [58]. The compound, named CLH304a, contains the same scaffold, di-(-*tert*-butyl)phenol, as CGP7930 and were found to be selective for the GABA_B_-R when tested in other class C members and decreased the IP_3_ production by GABA-induced G-protein activity. The NAM was used as starting point for developing derivatives, but pharmacological profiling showed problematic pharmacokinetics and toxicological profiles [83].

In general, the conserved nature of orthosteric binding sites between GPCRs, especially within subtypes, makes it challenging to obtain ligands with high selectivity, as seen for several family A GPCRs and the mGlus. Allosteric modulation may than be an alternative for increasing the selectivity [106]. However, the GABA_B_-R is the only metabotropic GABA receptor containing only one GABA binding site, and the main purpose for obtaining allosteric GABA_B_-R modulators is not necessary for increasing selectivity, but more important for enhancing beneficial pharmacological effects by reducing or potentiating the effects of GABA.

Development of allosteric modulators is challenging. The binding modes and molecular mechanisms of allosteric modulators are still quite uncertain, as previously discussed, and multiple allosteric binding sites may exist. The measurable functional effect of an allosteric modulator might also depend on the affinity of the compounds and the orthosteric ligand used, which also may complicate the screening procedures due to putative biased signaling. Binding of an allosteric modulator can contribute to stabilization of a receptor conformations induced by the agonist and thereby contribute to activation of a specific signaling pathway, unique for the specific ligand combination [64,66,107]. It must also be noted that the allosteric binding site is not as highly conserved between species as the orthosteric site, and therefore species-specific differences may affect the result of testing of potential drug candidates in animal models [55].

## 7. Recent and Future Advancements in GABA_B_ Receptor in Silico Drug Discovery

Advances in structural biology the past 20 years have increased the number of solved GPCR 3D structures tremendously. The first import breakthrough came in the year of 2000 with the release of the X-ray structure of bovine rhodopsin [108], and developments in imaging techniques during the last years, including cryo-EM [109], have given new opportunities within the field. The number of solved GPCR structures has dramatically increased since the release of the bovine rhodopsin structure with 346 currently resolved structures of the 7TM domain (data from the G protein-coupled receptor database (GPCRdb)), April 2020). In total, 85 of 346 solved complexes are active-state receptors.

Conventional drug discovery methods rely on stepwise synthesis followed by multiple assays for screening a large number of compounds to identify potential drug candidates [110]. The last decades, efforts have been put into in silico drug discovery strategies for drug design and screening, which represent more cost- and time-effective methods compared to the more conventional methods. Until recently, only 3D structures of the GABA_B_-R orthosteric binding site were available in the PDB-database, limiting the use of structure-based drug discovery to the orthosteric site. Investigations of the allosteric binding pocket have been restricted to homology models [57], which is a construct of a protein 3D structure based on the amino acid sequence and homologues proteins with known 3D structures [110]. The recent cryo-EM structures of the full receptor in both active and the inactive conformational states, may provide new possibilities in in silico drug discovery and dynamic characterization of the GABA_B_-R. A handful in silico ligand development studies targeting the GABA_B_-R orthosteric pocket have been published in recent years. These studies have not succeeded in obtaining clinical compounds, but with available 3D structures of the full receptor, new possibilities are provided also for new orthosteric compounds. Most of the in silico studies have been a combination of ligand based and structure based methods to identify potential hits and explore the VFT mechanisms [52,53,111,112].

As an experimentally solved structure of a protein by methods such as X-ray crystallography represents a rigid conformation, the structure will not necessarily represent the dynamic equilibrium. The structure can be deformed and modified in the crystallization procedure, ending up being somewhat different from the structure in its native environment [113]. Recent improvements in hardware and software have made more extensive calculations like molecular dynamics (MD) implementable in structure-based drug discovery. MD simulations do not suffer from the static representation of GPCRs, and can be used to provide multiple conformations of a target and thereby introduce receptor flexibility into docking procedures, calculations of kinetic profiles associated with the binding process, and post processing of ligand–receptor complexes in addition to multiple other purposes [114]. As MD is limited to, at most, a millisecond range, important events such as GPCR activation cannot be fully explored. Major conformational changes are also often separated by high energy barriers. Enhanced sampling methods including metadynamics and steered MD are becoming more popular to speed up slow processes and accelerate rare events that normally is inaccessible by unbiased MD [114]. Metadynamics-based protocols for calculating binding affinity and transition state ensemble for GPCRs have recently been published for class A and is under development for class B GPCRs, and represent methods of major importance for future in silico GPCR drug design and discovery [113,115].

## 8. Conclusion

The GABA_B_-R is involved in a broad range of diseases, and the receptor is considered a highly interesting therapeutic target. However, at present only one compound is in clinical use. Identification of allosteric modulators may increase the therapeutically potential of GABA_B_-R compounds. The recent cryo-EM structures of the full receptor show similarities to other class C GPCRs, and give new possibilities in the development of compounds. Further characterization and investigation of putative allosteric binding pockets, intracellular signaling pathways and development of new ligands are important for fully understanding the receptor-mediated mechanisms and pathophysiology, and for developing new drugs with favorable pharmacokinetics for clinical use.
